# A study on the genus *Candolleomyces* (Agaricales: Psathyrellaceae) from Punjab, Pakistan

**DOI:** 10.1186/s12866-023-02938-2

**Published:** 2023-07-11

**Authors:** Muhammad Haqnawaz, Abdul Rehman Niazi, Abdul Nasir Khalid

**Affiliations:** Fungal Biology and Systematics Research Laboratory, Institute of Botany, University of the Punjab, Quaid-e-Azam Campus 54590, Lahore, Pakistan

**Keywords:** Macrofungi, Kot Addu, Systematics, DNA barcoding, Punjab

## Abstract

Many basidiomata of the genus *Candolleomyces* were found on sandy and loamy soil from the Indus Riverbed, Kot Addu District. A phylogenetic study was conducted to examine the occurrence of *Candolleomyces sindhudeltae* sp. nov. using a combination of ITS and LSU regions. Our morphological, anatomical, and phylogenetic studies indicated the novelty of *Candolleomyces sindhudeltae* sp. nov. The distinguishing features of *C. sindhudeltae* are convex to campanulate and areolate pileus with scalloped to cracked cap margins, branched, and pale reddish lamellae, greenish-brown ellipsoid to ovoid basidiospores, polymorphic cheilo, and caulocystidia. The novel taxa formed independent phylogenetic relationships within the genus *Candolleomyces*. The addition of our new species to the genus *Candolleomyces* makes us confident that the genus was separated from *Psathyrella* accurately.

**Supplementary Information:**

The online version contains supplementary material available at 10.1186/s12866-023-02938-2.

## Introduction

The genus *Candolleomyces* D. Wächt. & A. Melzer (Psathyrellaceae) is characterized by small to medium-sized and dark spored mushrooms with brittle, fragile basidiomata, veil most likely always present but often very fugacious. It is differentiated from other closely related genera based on the absence of pleurocystidia. These can grow terrestrially, lignicolously, or rarely fimicolously and have small to large basidiomata [1–4]. Recently Wächter & Melzer transferred only 26 species into the genus *Candolleomyces*. Thirty-two (32) species worldwide have been recorded Asia is the richest with diversity of genus *Candolleomyces* having thirteen species with eleven from Europe, six from North America, and one species each from Africa, and South America.

From Pakistan, three species, *C*. *candolleanus* (Fr.) D. Wächt. & A. Melzer, *C*. *efflorescence* (Sacc.) D. Wächt. & A. Melzer [5] and *C. asiaticus* M. Asif, A. Izhar, Niazi & Khalid have been previously reported [2]. Many basidiomata of the genus *Candolleomyces* in a field study at the bed of the Indus River, Kot Addu, that previously undescribed. Morpho-anatomical characters and molecular phylogenetic analyses of nrITS and LSU sequences have been done to ascertain their position.

## Results

### Phylogenetic analysis using ITS region

#### ITS phylogenetic analyses (Fig. 3)

The phylogenetic tree of the ITS region consists of 40 sequences, as ingroup, and *Psathyrella thujina* A.H. Sm. (KC992876) as an outgroup. Our new taxon is shown in bold in the phylogenetic tree. *C. sindhudeltae* (OQ247908) formed a separate clade with 100 bootstrap values from its near species *C*. *tuberculatus* (KC992934) with about 25 bp differences. This clad contain three other speceis *C. leucotephrus* (MF325979), *C. efflorescens* (KC992941) and *C. albipes* (KX017209). In the sister clade nearest species *C. cladii-marisci* (MK080112) described. Our sequence data differs from other species of *Candolleomyces* available in GenBank. The phylogenetic analysis distinguished it from closer ones, especially *C. albipes*, our taxon is proposed as a new species within the genus *Candolleomyces*.

#### ITS & LSU phylogenetic analyses (Fig. 4)

The final phylogenetic tree of the combine ITS and LSU regions consists of 28 sequences as ingroup, and one sequence of *Psathyrella thujina* (KC992876) was chosen as an outgroup. The phylogenetic tree shows our two taxa, *C*. *sindhudeltae* as bold. The sequences of our taxon do not match the sequence data for other species of *Candolleomyces* available in GenBank. Our new taxa formed a separate branch from their nearest species with strong bootstrap value.

### Taxonomy

#### ***Candolleomyces sindhudeltae*** Haqnawaz, Niazi, & Khalid *sp. nov.* (Figs. 1 and 2)

**Fig. 1:**
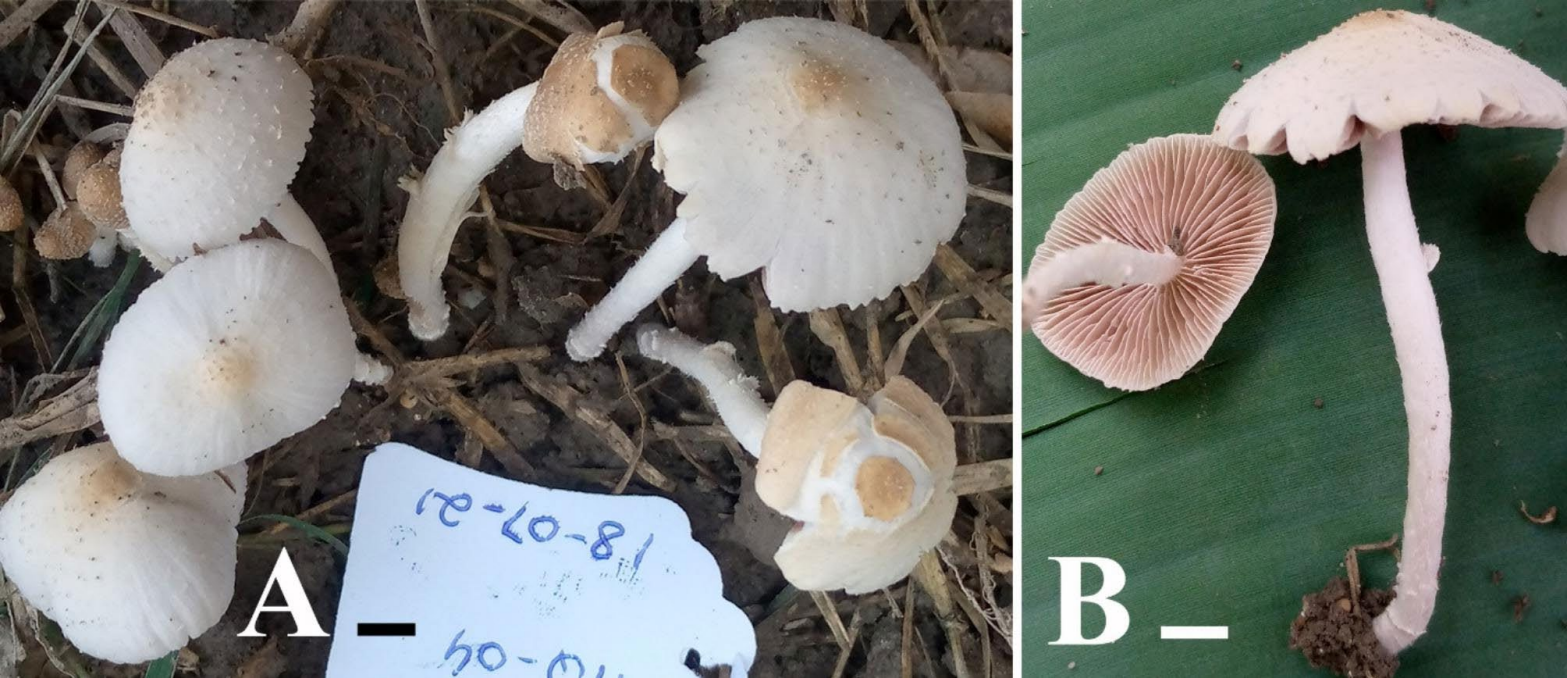
** A–B.** Basidiomata of *Candolleomyces sindhudeltae*, **B** = Holotype Scale bars: **A**–**B** = 10 cm. Photos by: Muhammad Haqnawaz

**Fig. 2:**
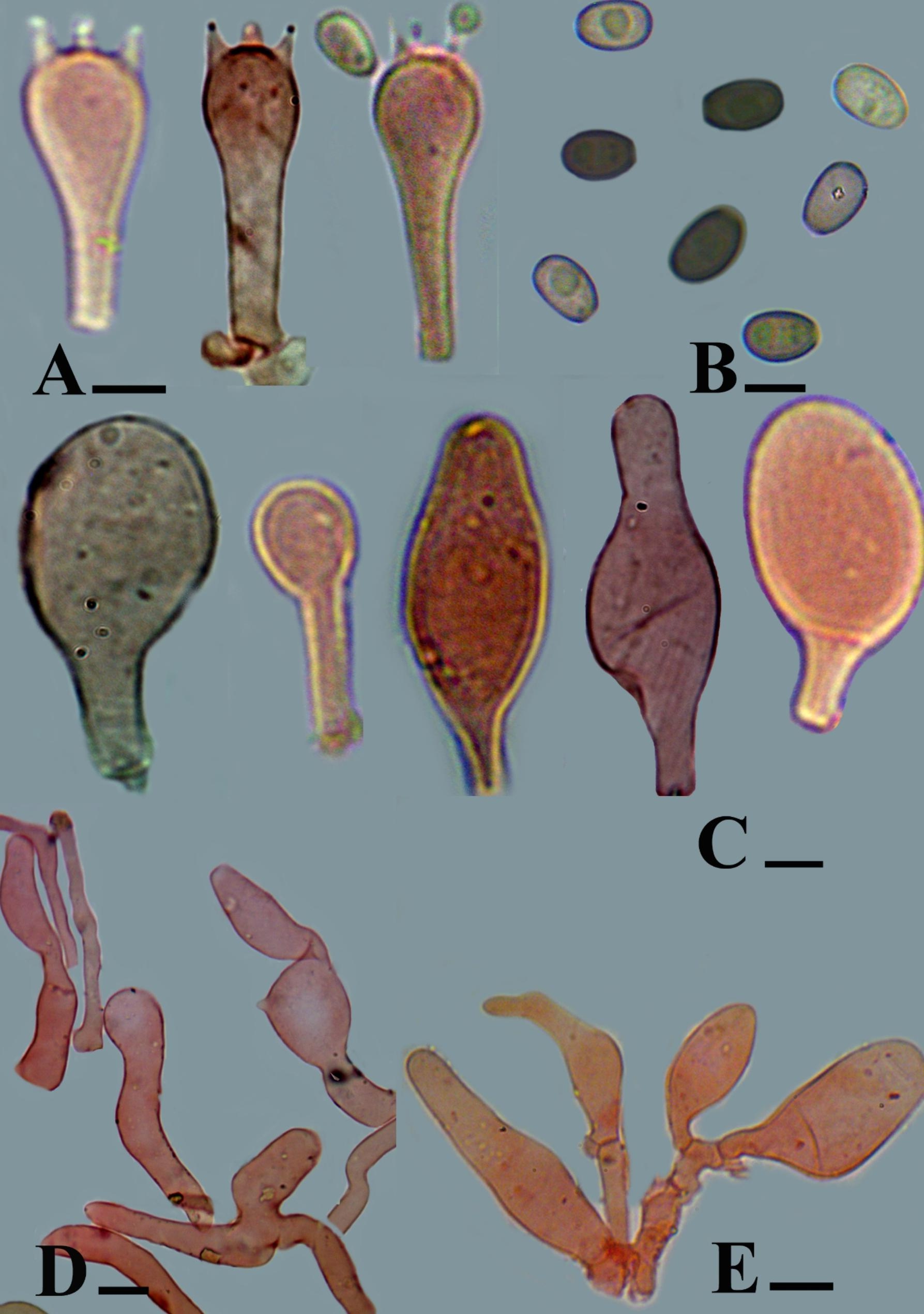
** A − E.** Microscopic structures of *Candolleomyces sindhudeltae* (Holotype. LAH37632). **A**. Basidia, **B**. Basidiospores, **C**. Cheilocystidia, **D**. Pileipellis elements, **E**. Caulocystidia, Scale bars: **A**–**C** = 5 μm, **C** = 30 μm, **D** = 10 μm. Photos by: Muhammad Haqnawaz

**Fig. 3:**
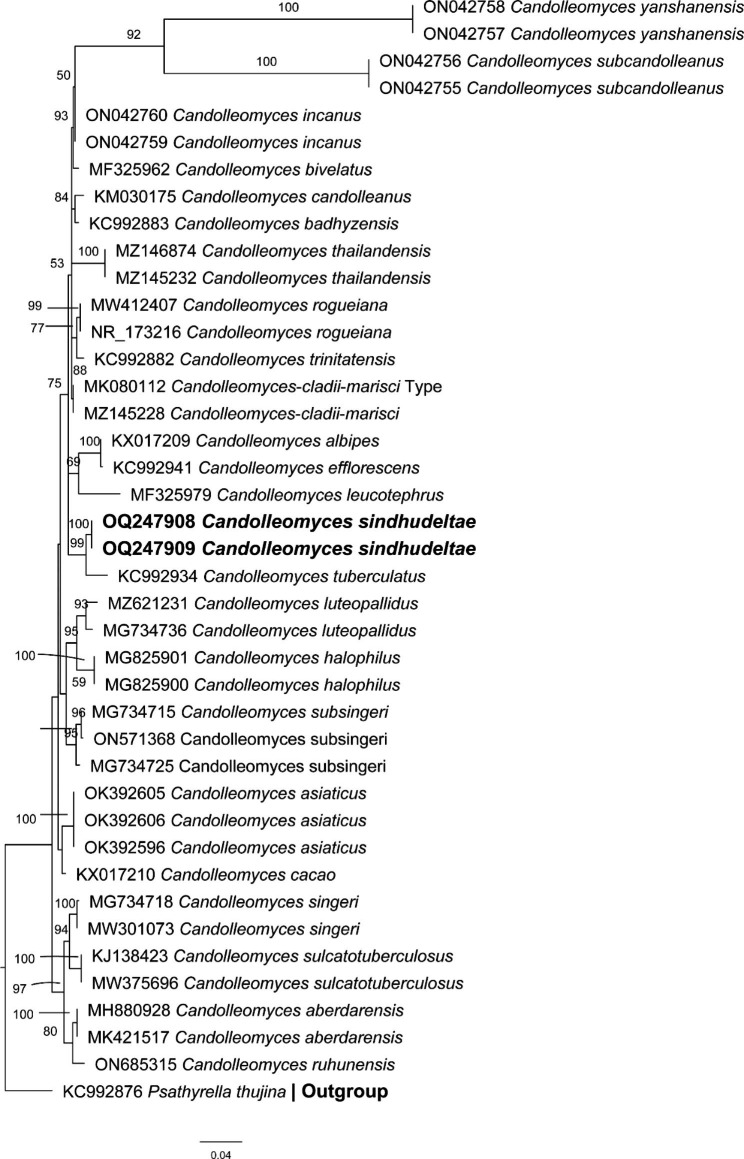
Molecular phylogenetic analyses of *Candolleomyces* species by maximum likelihood (ML) method based on ITS sequences. The evolutionary history was inferred by using the RAxML-HPC2 v 8.1.11. The analyses involved 40 nucleotide sequences and new species is in bold. Evolutionary analyses were conducted in CIPRES Portal v. 3.1. bootstrap values > 50% based on 1000 replicates are shown above the branches

**Fig. 4:**
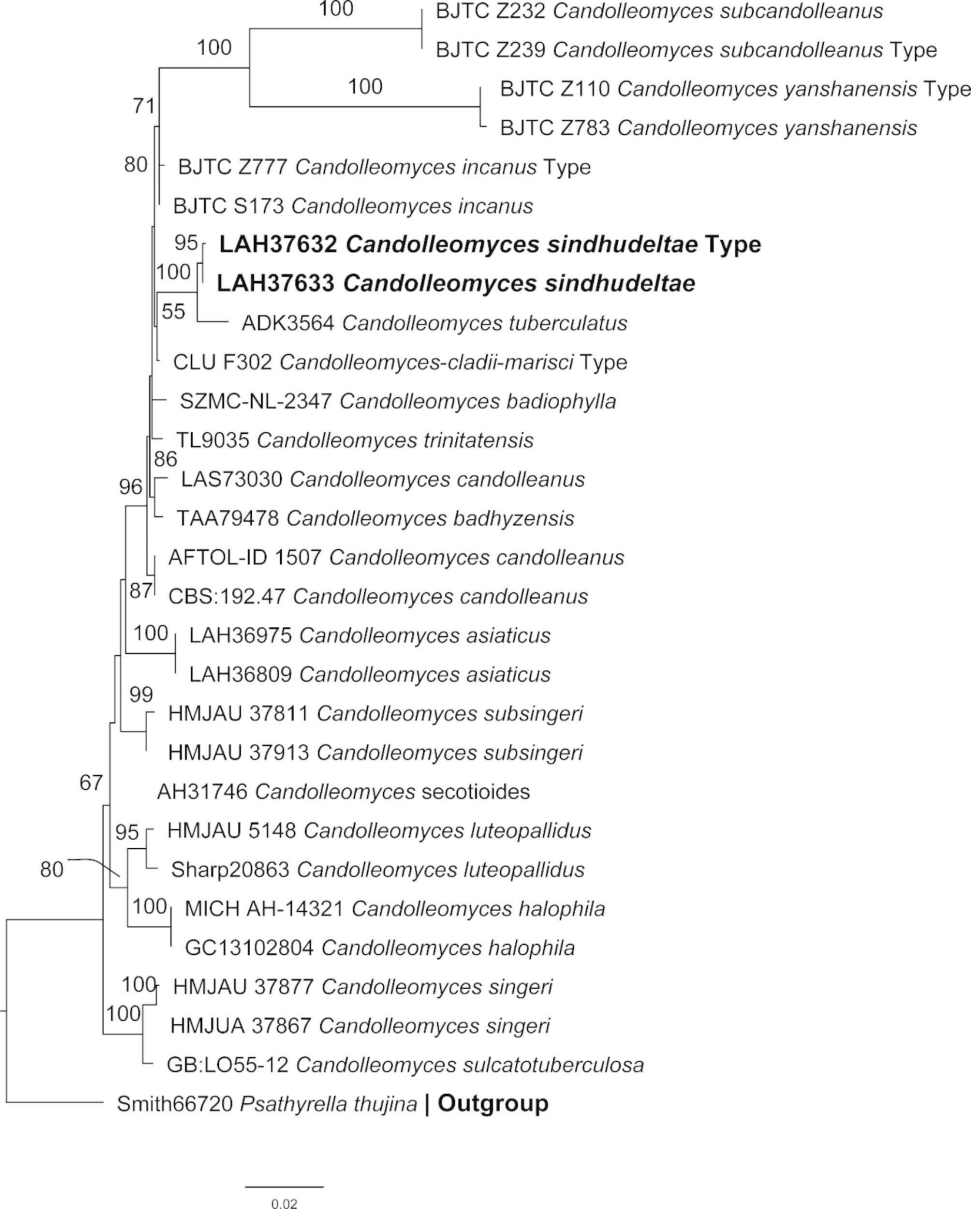
Molecular phylogenetic analyses of *Candolleomyces* species by maximum likelihood (ML) method based on combined ITS & LSU sequences. The evolutionary history was inferred by using the RAxML-HPC2 v 8.1.11. The analyses involved 28 nucleotide sequences and new species are in the old. Evolutionary analyses were conducted in CIPRES Portal v. 3.1. Bootstrap values > 50% based on 1000 replicates are shown above the branches

MycoBank MB847164.

Etymology:**—**Species name *sindhudeltae* (Latin) refers to the type locality of the taxon bed of the Indus River, Kot Addu, Punjab, Pakistan.

Diagnosis:— Convex to campanulate, dull yellow orang veils and areolate pileus with scalloped to cracked cap margins, branched and pale reddish lamellulae, light gray flexuous stipe with squamules, greenish-brown basidiospores, moderately clavate to broadly clavate, capitate, ligniform, globose, narrowly utriform to utriform, rarely ovoid pedunculate cheilocystidia, polymorphic elements of pileipellis 36–104 × 9–41 μm, presence of cylindrical, clavate, ovoid, oblong, utriform, and rarely ellipsoid, 15.79–42.7 × 7.57–16 μm caulocystidia.

Holotype:—Pakistan. Punjab, Kot Addu district, Indus Riverbed, Mauza Pirhar Gerbi, Lang Walla (30°25’21” N, 70°52’46"E, 132 m a.s.l.) on soil, rich in organic matter, July 18, 2021, *Muhammad Haqnawaz*, (LAH37632), GenBank OQ247908 (ITS), OQ247912 (LSU).

Description:— Pileus 13–40 mm diam., areolate, bilayer, parabolic to campanulate, slightly umbonate at a young stage and convex to campanulate when old, dull yellow-orange (10 YR 7/4) upper layer with light gray (10 YR 8/1) veil elements. Dull yellow-orange (10 YR 7/4) at the center and grayish white (N 8/0) to light gray (7.5 YR 8/1) toward the margin and light gray (7.5 YR 8/1) squamules. Decurved shape and scalloped to cracked cap margins, and not hygrophanus. Lamellae free, 3–4 tires, broad, average, close, unequal, branched, serrate, light gray (5 YR 8/1) at immature and pale reddish (2.5 YR 7/3) when mature. Stipe 20–35 mm, light gray (7.5 YR 8/1), central, flexuous, squamules present of the same color, Annulus ring-like, and Volva absent.

Basidiospores [20/2/2], (5.2–)5.5–7.0 (–7.1) × (3.4–)3.7–4.7(–4.6) µm, Q = 1.2–1.7, Qav = 1.56, ellipsoid to ovoid, greenish brown in 5% KOH, inamyloid thick–walled, smooth, prominent germ pore 1–2 μm, guttulate. Basidia (18–)18.3–28.1(–28.97) × (5.6–)5.8–7.9 (–8.38) µm, narrowly clavate to clavate, hyaline in 5% KOH, thick–walled, smooth, with 1–4 sterigmata, basal clamp connection absent. Cheilocystidia (13.08–) 15.8–35.1(–37.6) × (8.2–) 8.6–13.6(–15.4) µm, moderately clavate to broadly clavate, capitate, ligniform, globose, narrowly utriform to utriform, rarely ovoid pedunculate, thick-walled, hyaline in 5% KOH. Pleurocystidia absent. Pileipellis trichoderm hyphae, 6.6–15.6 μm diam, polymorphic elements, 36–103.9 × 8.5–41.4 μm, hyaline to yellowish in 5% KOH, thin-walled but the anatomy of both layers have little difference in shapes. Stipitipellis subregular hyphae, branched 4.3–22.4 μm diam., hyaline in 5% KOH, thin–walled septate, and clamp connection present. Caulocystidia cylindrical, clavate, ovoid, oblong, utriform, and rarely ellipsoid, 15.79–42.7 × 7.57–16 μm, hyaline in 5% KOH, clamped.

Ecology: Gregarious and caespitose, terrestrial, on loamy soil rich in organic matter.

Material examined: Pakistan, Punjab, Kot Addu, bed of Indus River, Safari Park (Lashari Wala Jungle), 30°28’33” N, 70°53’26"E, 134 m a.s.l.), on loamy soil rich in organic matter, August 11, 2022, *Muhammad Haqnawaz*, (LAH37633). GenBank OQ247909 (ITS) & OQ247913 (LSU).

## Discussions

*C. sindhudeltae* is described by macro-morphological, microscopic, and ITS rDNA characters to unveil the *Candolleomyces* diversity in Pakistan. The species has unique characteristics as shown in Table 1. The new species *Candolleomyces sindhudeltae* distinguishes from sister species *C. tuberculatus* by its globose, and tubercles scales of pileus, blakish brown stipe with membranus annulus and purple ovoid basidiospores [6]. Similarly *C*. *leucotephrus* differ due to its caespitose, pale to whitish color, the wrinkled surface of the pileus, and silky fibrillose stipe [7]. *C*. *efflorescens* has hemispheric pileus covered with saccharine matter and brown purple basidiospores [8] and *C*. *albipes* by its areolate surface with squamules, campanulate, scalloped to cracked cap margins of pileus, presence of annulus. *Candolleomyces albipes*, has glabrous, incurved to the recurved margin of pileus, and *C. cladii-marisci* has hemispheric to applanate pileus, and absence of annulus [9].

**Table 1:** Characteristics distinguishing *Candolleomyces sindhudeltae* from the other *Candolleomyces* species

Species name	Pileus	Pileus margin	Lamellae	Cheilocystidia	Caulocystidia	Pileipellis elements size
***C. albipes***	Convex to broadly convex	Incurved to recurved	Adnate, unbranched, pale grayish brown	Broadly clavate, 19–24 × 9–13.5 μm	Absent	10–30 μm in diam.
***C. badhyzensis***	Broadly bell-shaped, glabrous	No data	Whitish	Polymorphic, 34–51 × 8.5–15 μm	Absent	No data
***C. cladii-marisci***	Hemispheric to applanate	Deeply striate	Adnate, unbranched, Brown purplish	Versiform, utriform, seldom cylindrical to clavate,	Absent	No data
***C. incanus***	Hemispherical to conical, incanus to nude		Adnate, off-white	Utriform, apex obtuse or broadly obtuse or often subcapitate, rarely with deposits, 17–27 (31) × 7–11 (13) µm		25–32 μm broad
***C. sindhudeltae***	Convex to campanulate, areolate	scalloped to cracked	Free, branched, pale reddish	Clavate to broadly clavate, capitate, ligniform, globose, narrowly utriform to utriform, rarely ovoid pedunculate, 13–38 × 8–15 μm	Present	36–103.9 × 8.5–41.4 μm

Anatomically our new taxa *C. sindhudeltae* has the strongly variable shapes of the cheilocystidia (capitate, ligniform, globose, and rarely ovoid pedunculate) and the caulocystidia (cylindrical, ovoid, oblong, and rarely ellipsoid) shows the strong uniqueness of our taxon. but in *C. albipes* has only one shaped (broadly clavate) cheilocystidia and the absence of caulocystidia. *C. cladii-marisci* has versiform seldom cylindrical cheilocystidia and absence of caulocystidia. Sphaeropedunculate caulocystidia of *Candolleomyces aberdarensis* shows uniqueness of our new taxon [10].

*Candolleomyces sindhudeltae* shows some similarities with *C*. *caespitosus* and *C*. *paecilospermus* by having convex pileus, absence of pleurocystidia and ellipsoid basidiospores. But presence of polymorphic cheilocystidia, ring like of annulus, areolate surface of *C*. *sindhudeltae* differentiate it from *C*. *caespitosus* has flucose scale, absence of annulus. Moreover *C*. *paecilospermus* has broadly bell-shaped and flocculent pileus, clavate or utriform cheilocystidia. On comparing *Candolleomyces* with *Psathyrella* subsection Flocculosa these are similar due to lacking pleurocystidia. Similarly *Psathyrella flocculosa* show presence of veil. However it differ as follow: *P. flocculosa* has wooly tuft, absence of annulus, elliptic to ovate basidiospores, and subcylindrical to ventricose cheilocystidia. *C*. *sindhudeltae* has squamules on pileus, presence of annulus, ellipsoid to ovoid basidiospores, polymorphic cheilocystidia [11].

Occurrence of veil is a distinguish feature of genus *Candolleomyces* and absence of pleurocystidia is similar feature of *Candolleomyces* and *Psathyrella* in section Subtratae, but rarely veil present in some species of sect. Subtratae like *Psathyrella rudericola* A.H. Sm., *P*. *agerestis* A.H. Sm., and *P*. *sepulcreti* A.H. Sm. *Psathyrella rudericola* is differ from (*C*. *sindhudeltae* sp. nov.) due to its glabrous surface, absence of annulus, elliptic to oblong basidiospore, fusoid ventricose with obtuse apex cheilocystidia, and rarely subcylindrical to clavate caulocystidia. *Psathyrella agerestis* differ from our taxon due to its glabrous surface, absence of annulus, elliptic to ovate basidiospores and clavate to saccate cheilocystidia. *P*. *sepulcreti* shows the difference from new taxon by having obtusely umbonate, conicus, fluffy fibrils at base of stipe, absence of annulus, ovate to elliptic basidiospore, and absence of caulocystidia. *C*. *sindhudeltae* differ from the above *Psathyrella* sect. Subtratae by having squamules, areolate surface, ring-like annulus, ellipsoid to ovoid basidiospores, and polymorphic cheilocystidia and caulocystidia [12].

## Materials and methods

### Sampling site

During surveys of the fungal field of several places of District Kot Addu, the Indus Riverbed, Punjab in the rainy season from July to December, (2020-22) (30°25’21” N, 70°52’46"E, 132 m a.s.l.). We collected samples of genus *Candolleomyces* from different localities bed of the Indus River. The climate of the Kot Addu district is very hot in summer and mild in winter, the approximate highest temperature is 51 °C and the lowest temperature is minus 1 °C; the average annual rainfall is 127 mm [13].

The soil texture is sandy loam and suitable for a variety of crops. The predominant flora consists of *Vachellia nilotica* (L.) P.J.H.Hurter & Mabb, *Saccharum bengalense* Retz, *Dalbergia sissoo* Roxb, *Tamarix aphylla* (L.) H. Karst., *Saccharum spontaneum* Linn. and *Calotropis procera* (Ait.) Ait. F [12].

### Morpho-anatomical observations

Specimens were photographed and morphological features of fresh basidiomata were recorded. Micro-morphological characters of samples were described following Vellinga [14]. Color codes were from the Munsell Color Chart [15]. The collection was brought to the laboratory and dried under a fan heater. The dried sample was packed in polythene bags, kept in -80 °C refrigerators for preservation, and deposited in the LAH Herbarium, Institute of Botany, University of the Punjab, Quaid–e–Azam Campus, Lahore, Pakistan. For the microscopic study, tissues from different parts of the basidiomata were rehydrated in 5% KOH. Where necessary, tissues were stained in Congo Red (1%) and Melzer’s reagent. Microscopic structures were examined using a compound microscope (OLYMPUS U–DA 2E16295) and measured using ScopeImage9.0 (H9D).exe software connected to the microscope (CX_RII_) through a digital camera (HDCE–90D).

The short form [n/m/p] indicates ‘n’ basidiospores measured from ‘m’ basidiomata of ‘p’ collections. Basidiospore measurements were recorded as (a–) b–c (–d), where a = extreme minimum value, range b–c covers at least 90% of the calculated values, and d = extreme maximum value, ‘Q’ shows the individual spore length/width ratio while ‘Qav’ presents the average of all Q values.

### Molecular phylogeny and phylogenetic analyses

The DNA was extracted from dried samples of lamellae followed by a 2% modified CTAB method [16] then DNA was confirmed by Gel electrophoresis. A polymerase chain reaction was carried out for the amplification of the Internal Transcribed Spacer (ITS) region of the nuclear ribosomal DNA using the forward ITS1F and the reverse primer ITS4 [17]. The PCR cycling to amplify the ITS region was followed by Izhar [18] i.e. 5 min for denaturation at 95 °C followed by 35 cycles of annealing at 94 °C (1 min), 1.5 min at 55 °C, 1.5 min at 72 °C and a final extension at 72 °C for 5 min. LROR and LR5 were used to amplify the Large Sub Unit (LSU) region of fungal DNA [19]. For PCR of the LSU region, initial denaturation at 94 °C for 2 min, 35 cycles at 94 °C for 1 min, 52 °C for 1 min, 72 °C for 1 min, and final extension at 72 °C for 7 min. The PCR products were then sequenced. The final ITS and LSU sequences were obtained by assembling a consensus from both forward and reverse primer reads using BioEdit v 7.2.5 [20]. NCBI BLAST searched and retrieved nucleotide sequences from the GenBank database. The sequences with the closest identity were selected from BLAST results. Some closely related species from published data were added to the final dataset. The final maximum likelihood phylogenetic trees of ITS and LSU regions were constructed using RAxMLHPC2 on XSEDE tool (8.2.10) in the Cipres portal with 1000 bootstrap replicates and were visualized in FigTree v. 1.4.2 [21]. The newly generated sequences were deposited in GenBank and are in bold in the phylogenetic trees.

## Electronic supplementary material

Below is the link to the electronic supplementary material.

Supplementary Material 1

Supplementary Material 2

Supplementary Material 3

## Data Availability

The data has been submitted to GenBank and will be made available prior to manuscript publication. The accession number of the holotype are OQ247908 (ITS), OQ247912 (LSU) and paratype are OQ247909 (ITS) & OQ247913 (LSU). The data used in this study is available from the corresponding author on request.
