# Supplementary material for: A study on the genus Candolleomyces (Agaricales: Psathyrellaceae) from Punjab, Pakistan
Source: BMC Microbiol. 2023 Jul 11;23:181. doi: 10.1186/s12866-023-02938-2 (PMC10334618; doi:10.1186/s12866-023-02938-2)
Supplement: Supplementary file 1 — Supplementary Material 1 [file 12866_2023_2938_MOESM1_ESM.pdf]

Candolleomyces sindhudeltae

Classification: *Fungi, Dikarya, Basidiomycota, Agaricomycotina, Agaricomycetes, Agaricomycetidae, Agaricales, Psathyrellaceae, Candolleomyces*

Summary: *Candolleomyces sindhudeltae Haqnawaz, Niazi, & Khalid, Pl. Syst. Evol.: 23 (2023) [MB#847164]*

Epithet: *sindhudeltae*

Type of name: Basionym

Basionym:

Author(s): Haqnawaz, Niazi, & Khalid

Year: 2023

Name status: Legitimate

MycoBank #: 847164

Remarks:

Page: 23

Gender: Unknown

Rank name: sp.

Etymology: Species name sindhudeltae (Latin) refers to the type locality of the taxon bed of the Indus River, Kot Addu, Punjab, Pakistan.

Type name:

Description

Remarks: The addition of our new specie to the genus Candolleomyces makes us confident that the genus was separated from Psathyrella accurately.

Description: The addition of our new specie to the genus Candolleomyces makes us confident that the genus was separated from Psathyrella accurately.

Bibliography

Title: A study on the genus Candolleomyces (Agaricales: Psathyrellaceae) from Punjab, Pakistan

Authors: MUHAMMAD HAQNAWAZ, ABDUL REHMAN NIAZI AND ABDUL NASIR KHALID

Editors:

Year: 2023

Volume:

First page:

Last page:

Issue:

Keywords: Macrofungi, Kot Addu, Systematics, DNA barcoding, Punjab

Abstract: Many basidiomata of the genus Candolleomyces were found on sandy and loamy soil from the Indus Riverbed, Kot Addu District. A phylogenetic study was conducted to examine the occurrence of Candolleomyces sindhudeltae sp. nov. using a combination of ITS and LSU regions Our morphological, anatomical, and phylogenetic studies indicated the novelty of Candolleomyces sindhudeltae sp. nov. The distinguishing features of C. sindhudeltae are convex to campanulate and areolate pileus with striate margins, free, branched, and pale reddish lamellae, greenish-brown ellipsoid to ovoid basidiospores, polymorphic cheilo, and caulocystidia. The novel taxa formed independent phylogenetic relationships within the genus Candolleomyces. The addition of our new specie to the genus Candolleomyces makes us confident that the genus was separated from Psathyrella accurately.

DOI:

Journal: Plant Systematics and Evolution

Type Specimen Information

Type of specimen: Holotype

Specimens #: LAH37632

Collected by: Muhammad Haqnawaz,

Collectors #:

Collection date: 2021/07/07

Type specimen:

Host: no

Substrates: laomy soil

Habitat:

Isolated by:

Isolation method:

Sexual state: Punjab

Growth:

Sporulation:

Pathogenicity: Lahore
