# Supplementary material for: A study on the genus Candolleomyces (Agaricales: Psathyrellaceae) from Punjab, Pakistan
Source: BMC Microbiol. 2023 Jul 11;23:181. doi: 10.1186/s12866-023-02938-2 (PMC10334618; doi:10.1186/s12866-023-02938-2)
Supplement: Supplementary file 2 — Supplementary Material 2 [file 12866_2023_2938_MOESM2_ESM.pdf]

>OQ247908_Candolleomyces_sindhudeltae

TGGGGAATTAAAGTTGGRRAYKTMKRYTCGTAGKKGACCTGCGGAAGGATCATTAA

CGAATATCTATGGCGTTGGTTGTAGCTGGCTCCTAGGAGCATCTTGTGCACACCCGT

CATTCATATCATCTTTCCACCTGTGAACCATGTGTAGGCCTGGATACCCCTCGCTTTG

GCAACAAAGCGGATGCAAGGATTGCTGTGTCGACAAGGCCGGCTCTCTTTGAATTTC

CAGGTTCTATGTCTTTTACACACCCCATTTGAATGATGTAGAATGTAGTCAATGGGC

TTTCACGCCTATAAAACACTATACAACTTTCAGCAACGGATCTCTTGGCTCTCGCATC

GATGAAGAACGCAGCGAAATGCGATAAGTAATGTGAATTGGCAGAATTCAGTGAAT

CATCGAATCTTTGAACGCACCTTGCGCTCCTTGGTATTCCGAGGAGCATGCCTGTTTG

AGTGTCATTAAATTCTCAACCTCACCAGTTTTGTAACGAGACAGGTGAAGGCTTGGA

TGTGGGGGTTTTGCAGGCTGCCTCAGTGCTGGTCTGCTCCTCTGAAATGCATTAGCG

AGCTCATGTTGAGCCTCCGTCTATTGGTGTGATAATTATCTACGCCGTGGATTGGAA

CTCATGCTTGCTTCTAACCGTCCGCAAGGACCAATTAACTTGACCAATTGAACTTCA

ATTCAGGWAGAATACCCGCYTAACTTAAACATAWYCAWAACCGGGAAAGAAA

> OQ247909_Candolleomyces_sindhudeltae

TGGGGAATTAAAGTTGGRRAYKTMKRYTCGTAGKKGACCTGCGGAAGGATCATTAA

CGAATATCTATGGCGTTGGTTGTAGCTGGCTCCTAGGAGCATCTTGTGCACACCCGT

CATTCATATCATCTTTCCACCTGTGAACCATGTGTAGGCCTGGATACCCCTCGCTTTG

GCAACAAAGCGGATGCAAGGATTGCTGTGTCGACAAGGCCGGCTCTCTTTGAATTTC

CAGGTTCTATGTCTTTTACACACCCCATTTGAATGATGTAGAATGTAGTCAATGGGC

TTTCACGCCTATAAAACACTATACAACTTTCAGCAACGGATCTCTTGGCTCTCGCATC

GATGAAGAACGCAGCGAAATGCGATAAGTAATGTGAATTGGCAGAATTCAGTGAAT

CATCGAATCTTTGAACGCACCTTGCGCTCCTTGGTATTCCGAGGAGCATGCCTGTTTG

AGTGTCATTAAATTCTCAACCTCACCAGTTTTGTAACGAGACAGGTGAAGGCTTGGA

TGTGGGGGTTTTGCAGGCTGCCTCAGTGCTGGTCTGCTCCTCTGAAATGCATTAGCG

AGCTCATGTTGAGCCTCCGTCTATTGGTGTGATAATTATCTACGCCGTGGATTGGAA

CTCATGCTTGCTTCTAACCGTCCGCAAGGACCAATTAACTTGACCAATTGAACTTCA

ATTCAGGWAGAATACCCGCYTAACTTAAACATAWYCAWAACCGGGAAAGAAA
