# Supplementary material for: A study on the genus Candolleomyces (Agaricales: Psathyrellaceae) from Punjab, Pakistan
Source: BMC Microbiol. 2023 Jul 11;23:181. doi: 10.1186/s12866-023-02938-2 (PMC10334618; doi:10.1186/s12866-023-02938-2)
Supplement: Supplementary file 3 — Supplementary Material 3 [file 12866_2023_2938_MOESM3_ESM.pdf]

>OQ247912_Candolleomyces_sindhudeltae

TATACATATCAATAAGCGRMGGARMAWRAACTAACAAGGATTCCCCTAGTAACTGCGAGTGAAGCGGGAAGAG
CTCAAATTTAAAATCTGGTGGTCTCTGGCCATCCGAGTTGTAATCTAGAGAAGTGTTACCCGCGTCGGACCGTGTA
TAAGTCTCCTGGAATGGAGCGTCATAGAGGGTGAGAATCCCGTCTTTGACACGGACTACCGAGGCTTTGTGGTAT
GCTCTCAAAGAGTCGAGTTGTTTGGGAATGCAGCTCAAAATGGGTGGTAAATTCCATCTAAAGCTAAATATTGGC
GAGAGACCGATAGCGAACAAGTACCGTGAGGGAAAGATGAAAAGAACTTTGGAAAGAGAGTTAAACAGTACGT
GAAATTGCTGAAAGGGAAACGCTTGAAGTCAGTCGCGTTGGCCGGGAATCAGCCTTGCTTTTTGCTTGGTGTACTT
TCTGGTTGACGGGYCAGCATCAGTTTTGACCGGTGGAAAAAGTTCAGGGGAATGTGGCATCTTCGGATGTGTTAT
AGCCCTTGTTCGTATACATCGGTTGGGACTGAGGAACTCAGCACGCCGCAAGGCCGGGTCTTTGACCACGTTCGT
GCTTAGGATGCTGGCATAATGGCTTTAATCGACCCGTCTTGAAACACGGACCAAGGAGTCTAACATGCCTGCGAG
TGTTTGGGTGGAAAACCCGAGCGCGTAATGAAAGTGAAAGTCGAGATCCCTGTCATGGGGAGCATCGACGCCCG
GACCTGACGTTTTCTGACGGCCCTGCGGTAGAGCATGTATGTTGGGACCCGAAAGATGGTGAACTATGCCTGAAT
AGGGTGAAGCCAGAGGAAACTCTGGTGGAGGCTCGTAGCGATTCTGACGTGCAAATCGATCGTCAAATTTGGGT
ATAGGGGCGAAAGACTAATCGAACCCATYYMRTAGTGGTCCCTGCGGT

>OQ247913_Candolleomyces_sindhudeltae

GARMAWRAACTAACAAGGATTCCCCTAGTAACTGCGAGTGAAGCGGGAAGAGCTCAAATTTAAAATCTGGTGGT
CTCTGGCCATCCGAGTTGTAATCTAGAGAAGTGTTACCCGCGTCGGACCGTGTATAAGTCTCCTGGAATGGAGCGT
CATAGAGGGTGAGAATCCCGTCTTTGACACGGACTACCGAGGCTTTGTGGTATGCTCTCAAAGAGTCGAGTTGTTT
GGGAATGCAGCTCAAAATGGGTGGTAAATTCCATCTAAAGCTAAATATTGGCGAGAGACCGATAGCGAACAAGTA
CCGTGAGGGAAAGATGAAAAGAACTTTGGAAAGAGAGTTAAACAGTACGTGAAATTGCTGAAAGGGAAACGCTT
GAAGTCAGTCGCGTTGGCCGGGAATCAGCCTTGCTTTTTGCTTGGTGTACTTTCTGGTTGACGGGYCAGCATCAGT
TTTGACCGGTGGAAAAAGTTCAGGGGAATGTGGCATCTTCGGATGTGTTATAGCCCTTGTTCGTATACATCGGTTG
GGACTGAGGAACTCAGCACGCCGCAAGGCCGGGTCTTTGACCACGTTCGTGCTTAGGATGCTGGCATAATGGCTT
TAATCGACCCGTCTTGAAACACGGACCAAGGAGTCTAACATGCCTGCGAGTGTTTGGGTGGAAAACCCGAGCGCG
TAATGAAAGTGAAAGTCGAGATCCCTGTCATGGGGAGCATCGACGCCCGGACCTGACGTTTTCTGACGGCCCTGC
GGTAGAGCATGTATGTTGGGACCCGAAAGATGGTGAACTATGCCTGAATAGGGTGAAGCCAGAGGAAACTCTGG
TGGAGGCTCGTAGCGATTCTGACGTGCAAATCGATCGTCAAATTTGGGTATAGGGGCGAAAGACTAATCGAACCC
ATYYMRTAGTGGTC
